# Identification of Antioxidant and Anti-Inflammatory Activity of Sea Cucumber (*Holothuria tubulosa*) Active Peptides by a Combined Approach of Omics Data and Bioinformatics Analysis

**DOI:** 10.3390/md24050158

**Published:** 2026-04-30

**Authors:** Laura La Paglia, Mirella Vazzana, Manuela Mauro, Francesca Dumas, Alfonso Urso, Sugár Simon, Laszlo Drahos, Aiti Vizzini

**Affiliations:** 1 Istituto di Calcolo e Reti ad Alte Prestazioni, Consiglio Nazionale delle Ricerche, Via Ugo La Malfa 153, 90146 Palermo, Italy; alfonso.urso@icar.cnr.it; 2 Dipartimento di Scienze e Tecnologie Biologiche, Chimiche e Farmaceutiche, Università di Palermo, Via Archirafi 18, 90100 Palermo, Italy; mirella.vazzana@unipa.it (M.V.); manuela.mauro01@unipa.it (M.M.); francesca.dumas@unipa.it (F.D.); 3 HUN-REN Research Centre for Natural Sciences, MS Proteomics Research Group, Magyar Tudósok Körútja 2, H-1117 Budapest, Hungary; sugarsimi@gmail.com (S.S.);

**Keywords:** peptides, inflammation, oxidation, bioinformatics, echinoderms, proteomics, artificial intelligence

## Abstract

Background: Inflammatory signaling and oxidative stress machinery are interconnected and play roles in apoptosis, proliferation, redox state control, and the progression of many diseases, including cancer. The marine environment harbors a wealth of organisms that produce a wide variety of bioactive molecules with significant biological activities. Over the last decade, the advent of AI-driven approaches has enhanced the study and analysis of peptides, helping to reduce costly and time-consuming conventional laboratory testing, validation, and synthetic procedures. Methods: In this study, we predicted the antioxidative and anti-inflammatory activities of peptides isolated from proteomic data obtained from circulating cells and humoral components of the sea cucumber defense system using a bioinformatic workflow based on different artificial intelligence tools. Results: We identified 40 top-ranked peptides with antioxidative and anti-inflammatory activity and a sub-class of eight peptides shared by FreD domains. Molecular docking and molecular dynamics simulations showed that they have active binding sites for different key molecules involved in inflammatory and oxidative processes. Conclusions: The results showed that the peptides highlighted by our analysis workflow can be identified as potential molecules used as therapeutic strategies for diseases by targeting both inflammatory and oxidative processes.

## 1. Introduction

Inflammation represents a complex protective mechanism through which the body responds to harmful stimuli, including pathogens, damaged cells, and foreign substances, with the goal of restoring physiological balance [1]. This process is initiated through the activation of various classes of pattern recognition receptors (PRRs) expressed on the surface of immune cells, such as C-type lectin receptors, Toll-like receptors (TLRs), or nucleotide-binding oligomerization domain-like receptors (NLRs) [2,3]. Upon activation, these receptors trigger intracellular signaling cascades that promote the production and release of cytokines, which further amplify and regulate the inflammatory response. These cytokines interact with diverse receptor families, including the tumor necrosis factor (TNF) receptor superfamily, the immunoglobulin superfamily, class I and II cytokine receptors, and chemokine receptors.

Engagement of PRRs activates multiple downstream signaling pathways, notably related to the intracellular signaling kinase cascade, including mitogen-activated protein kinases (MAPKs), phosphoinositide 3-kinase (PI3K), signal transducer and activator of transcription (STAT), and the IκB kinase (IKK)/nuclear factor kappa B (NF-κB) molecules, all of which coordinate the cellular response to inflammatory stimuli [4,5]. In addition to inflammation, oxidative stress is tightly interconnected with these signaling networks and has a central role in controlling processes such as apoptosis, cell proliferation, and redox homeostasis, which contribute to the development of different pathological conditions, such as cancer [6].

Oxidative stress starts when a disruption arises in the balance between the production of reactive species and the ability of antioxidant mechanisms to neutralize them. This imbalance promotes molecular damage, particularly affecting nucleic acids, proteins, and lipids, and can lead to altered cellular signaling and genomic instability [7,8]. Furthermore, the increase in oxidative damage over time has been linked to aging and the onset of age-related diseases. Reactive oxygen species (ROS), a major class of free radicals, are key mediators in this process and can impair normal cellular functions, ultimately contributing to inflammation, tissue injury, and organ dysfunction [9]. ROS exert their effects either through direct interaction with biomolecules or by modulating intracellular signaling pathways [10,11].

ROS are generated from both external and internal sources. Exogenous contributors include environmental factors such as radiation, pollution, smoking, infections, and exposure to chemicals or drugs, whereas endogenous production primarily occurs within cellular organelles such as mitochondria, lysosomes, and the plasma membrane. Enzymatic systems, including NADPH oxidases (NOX) and myeloperoxidase (MPO), are also involved in ROS generation and can induce structural modifications in proteins, DNA damage, and other cellular abnormalities. Given their high reactivity and potential toxicity, maintaining ROS at controlled levels is essential for cellular homeostasis and overall health [12].

To counteract oxidative damage, cells have evolved complex antioxidant defense mechanisms composed of both enzymes and non-enzymatic elements. Key antioxidants include the superoxide dismutase (SOD) and the catalase (CAT) enzymes. The former plays a major role in the conversion of superoxide radicals into hydrogen peroxide (H_2_O_2_). This last molecule is then detoxified into water and oxygen by different enzymes such as CAT, glutathione peroxidase (GPx), and peroxiredoxins (Prxs) [13,14]. Additionally, other enzymatic compounds like glutathione reductase (GR) and glutathione S-transferase (GST) contribute to maintaining redox balance through the regulation of glutathione metabolism [15].

ROS also influence several major inflammatory signaling pathways, including the NLRP3 inflammasome, MAPK, JAK/STAT, Nrf2, PI3K/AKT, and NF-κB pathways, thereby linking oxidative stress to inflammatory responses [16]. Among these, NF-κB is a pivotal transcription factor family that includes NF-κB1, NF-κB2, RelA (p65), c-Rel, and RelB, which regulate the expression of numerous genes involved in immune function, inflammation, cell survival, and proliferation [17]. NF-κB activation, often mediated through the degradation of IκBα, leads to increased production of pro-inflammatory cytokines, including interleukin-1β (IL-1β), IL-6, TNF-α, and transforming growth factor-β (TGF-β), as well as key enzymes such as inducible nitric oxide synthase (iNOS) and cyclooxygenase-2 (COX-2), along with their downstream mediators nitric oxide (NO) and prostaglandin E_2_ (PGE_2_) [18,19].

Given the central roles of inflammation and oxidative stress in disease progression, their regulation remains a major focus in biomedical research. Over the past decade, extensive efforts have been directed toward identifying natural compounds with antioxidant and anti-inflammatory properties [20,21,22]. Marine organisms can be an abundant source of bioactive molecules holding many pharmacological activities. Among them, the sea cucumber (*Holothuria tubulosa*), belonging to the class Holothuroidea of the phylum Echinodermata, has attracted considerable attention due to its production of biologically active metabolites exhibiting anticancer, antimicrobial, antioxidant, anticoagulant, and anti-inflammatory effects [23,24,25].

Notably, the bioactive metabolites triterpene glycosides (saponins), isolated from sea cucumbers, have demonstrated relevant anticancer potential across various experimental models. For example, frondoside A from *Cucumaria frondosa* has been extensively studied in breast, pancreatic, and prostate cancer models [26,27,28,29,30], while related compounds from *Mensamaria intercedens* have shown activity against lung cancer [31]. In addition, coelomocytes—immune cells present in the coelomic fluid of holothurians—play essential roles in host defense, stress response, wound healing, and tissue regeneration [32]. These cells exhibit functions such as phagocytosis, formation of “brown bodies,” cytotoxic activity, and secretion of antimicrobial and antifungal compounds, contributing to immune protection and tissue homeostasis [33,34,35,36,37,38,39,40,41,42,43].

Importantly, ROS exhibit a dual role depending on their concentration. At controlled levels, they act as signaling molecules involved in regulating inflammatory responses, whereas excessive ROS accumulation leads to oxidative damage of macromolecules, exacerbating inflammation and contributing to disease development [44]. Consequently, the development of bioactive peptides capable of modulating ROS levels represents a promising strategy for restoring redox balance and mitigating inflammation-induced damage.

Recent advances in artificial intelligence (AI) and bioinformatics have significantly accelerated peptide discovery and characterization, reducing reliance on time-consuming and costly experimental approaches. In this context, the present study aims to identify and characterize antioxidant and anti-inflammatory peptides derived from the circulating cells and humoral components of the sea cucumber defense system using an integrated proteomics and computational approach.

## 2. Results

**Figure 1:**
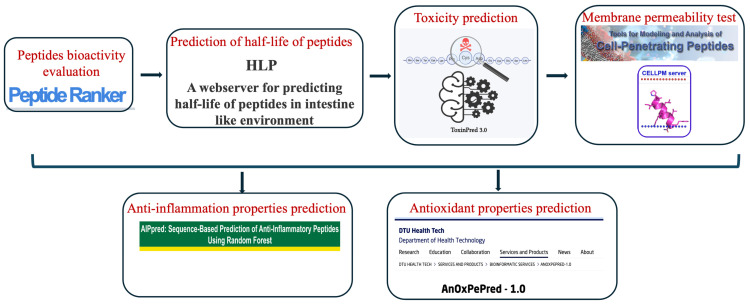
Bioinformatics analysis workflow.

We performed proteomic analysis of the humoral component (cell-free coelomic fluid) and the cellular component (cell pellet) of *H. tubulosa* coelomic fluid using mass spectrometry (MS), yielding 174 unique annotated proteins. Starting from 174 unique annotated proteins obtained from mass spectrometry, we applied a bioinformatic approach to select different peptides arising from protein data (via tryptic peptides). This allowed the production of computationally generated peptides with a 10-mer window from the identified protein sequences. These computationally derived peptides were then filtered through an in silico analysis involving different peptide features to rationalize and reduce the selection of candidates from the numerous sequences obtained through proteomics analysis. The bioinformatic approach used to yield a list of ranked peptides is shown in Figure 1.

First, we evaluated the bioactivity of the peptides using the web tool PeptideRanker, which uses a neural network algorithm, and selected the probability of bioactivity using a ranking score. We then analyzed the half-life of the peptides in the intestinal environment. We then analyzed the half-life of the peptides in the intestinal environment, as this feature is a relevant tasks when de-signing peptide-based drugs.

The susceptibility of a peptide to degradation by proteolytic enzymes is high, causing a rapid clearing from the body before it can perform or show the desired effect. This bioinformatics analysis filtered and deleted all peptides with lengths of ≤5 and ≥10 amino acids. The peptides that were candidates for having a long half-life in the intestinal environment were all 10 amino acids long. To detect half-life, we used the HLP tool and adopted stringent criteria. Only peptides with a stability value higher than 1.5 were considered for further analysis. As the final output of the workflow was the identification of candidate peptides to be used as new biological agents for human drug therapies, toxicity was also investigated. To this end, we used the ToxinPred tool, which is based on different machine learning models that are employed to classify a given peptide sequence by detecting the specific patterns present just in toxic and non-toxic peptides. We also investigated the cell membrane permeability of the peptides using the Cell-Penetrating Peptides server, which is part of the PerMM web tool. Finally, the anti-inflammatory and antioxidant features of the peptides were evaluated using the AIPred and AnOxPePred tools, respectively. These tools allow for the analysis of both the antioxidant group of free scavengers (FRS), which directly inhibit and quench free radicals, and the group of chelators, which cause a delay in the oxidation process by forming complexes with metal ions and blocking radical formation. Indeed, there are other forms of antioxidants, such as oxygen scavengers and antioxidant regenerators, but there is little experimental data available for these two groups; thus, we focused the analysis on the first two couples. The decision to analyze both anti-inflammatory and antioxidant properties relies on the evidence that they are deeply interconnected. This dynamic interplay is central to many chronic diseases; therefore, a dual strategy targeting both aspects will be more effective for prevention and treatment than focusing on only one aspect. At the end of the bioinformatics analysis workflow, we identified the top 40 peptides with all the previously discussed features (Table 1) (all detailed bioinformatics analysis results are in the Supplementary Material Excel sheet).

After selecting the top 40 peptides, we aimed to determine the protein to which they belong using functional annotation. Annotation analysis was performed using the UniProt web tool, as shown in Table 2. The 40 top-ranked peptides were selected because these proteins overcame all the threshold values applied for all the categories analyzed in the bioinformatics workflow.

**Figure 2:**
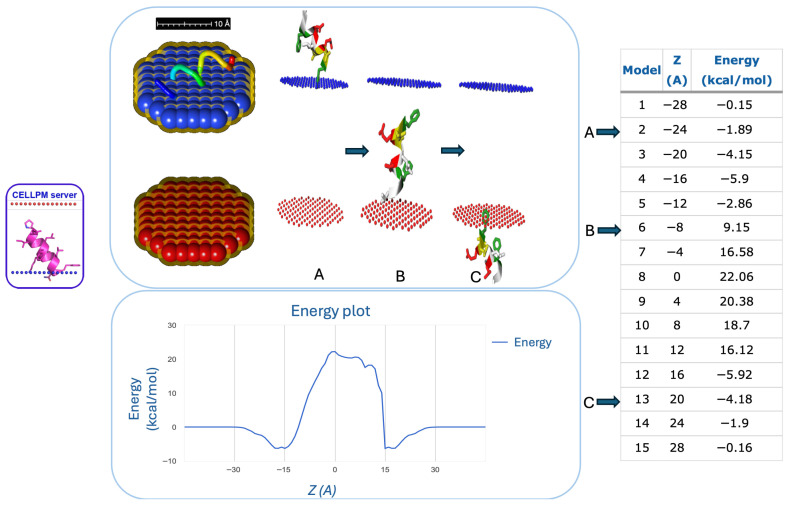
Left part of the figure: Membrane permeability through Cell-PM server of peptide VGFGDLNENF extracted from Ficolin-1 protein. Right part of the figure: The table indicates the energy used by the peptide to cross the cell membrane at different moments (from 1 to 15) and the Z (A) scores. The red and blue pseudo-atoms mark the hydrophobic boundaries of the lipid bilayer. A, B, and C are three different moments of cell membrane permeability of the peptide, with relative energy values. Lower part of the figure: The energy plot, on the lower part of the figure, is reported, highlighting the highest value used by the peptide to cross the cell membrane (20.38 kcal/mol).

As shown in the bioinformatics workflow (Figure 1), for all top-ranked peptides, we tested the permeability capacity to cross the cell membrane. Indeed, testing the permeability of a peptide across the cell membrane is crucial in drug development to ensure that therapeutic compounds can cross cell barriers, reach intracellular targets, and achieve the necessary bioavailability. Because peptides often have low passive permeability due to size and hydrophilicity, this testing helps optimize their design, such as through cyclization or modification, to avoid early-stage preclinical failures. The permeability test was performed through the PerMM web server, which allows the prediction and visualization of passive translocation of bioactive molecules across lipid membranes (Figure 2).

**Figure 3:**
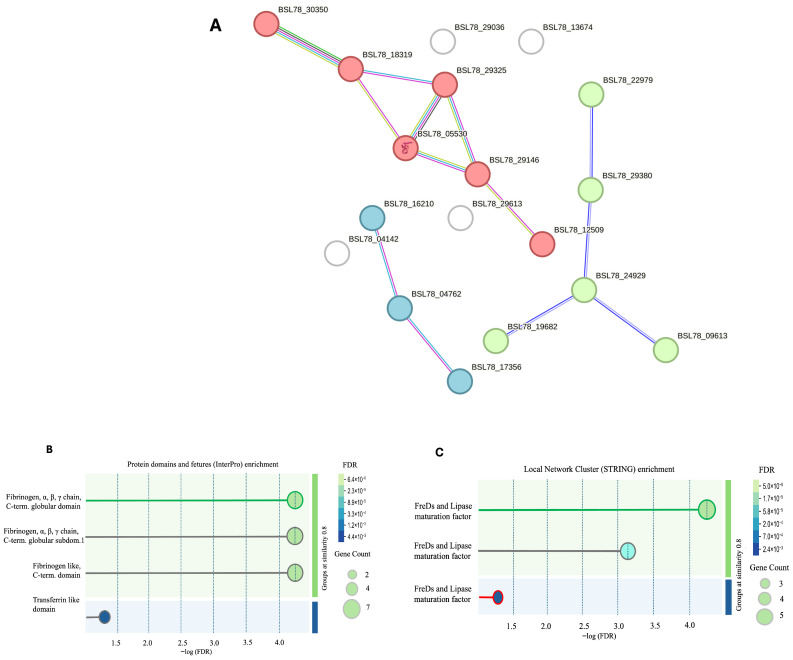
Clustering analysis of the putative annotated proteins to which selected peptides belong. (**A**): Clustering analysis was K-means clustering, and an FDR cut-off of <0.05 was applied to the analysis. (**B**): Protein domains and features enrichment by InterPro are shown. (**C**): Local network cluster enrichment. Analysis results are sorted by −logFDR (x axis) and similarity (y axis).

Once we identified the top-ranked anti-inflammatory and antioxidant peptides, we performed STRING analysis to identify potential interactions and functional analysis on proteins from which the peptides were derived, as it has been shown that processes like inflammation, oxidative stress and pathological conditions could have a common matrix. The STRING analysis results are shown in Figure 3. As shown in the figure, the clustering analysis performed on the selected proteins revealed three different clusters. The red cluster was formed by most proteins and had proteins like putative filamin-B, calmodulin-like, singed, Ras-related protein, and putative stomatin-like protein. The green cluster was linked to proteins of the fibrinogen family, and the blue cluster was linked to proteins linked to the transferrin family. A few proteins were not inserted in any cluster, namely techylectin-5B, putative ficolin-2-like, phospholipid scramblase, and putative nucleoline. Moreover, the functional enrichment analysis by STRING revealed molecular functions enriched for signaling receptor binding (GO:0005102). Moreover, local network clustering showed the presence of fibrinogen-related domains (FREDs) and lipase maturation factors. The FDR threshold was <0.05 for all the functional enrichment results, and a similarity score of ≤0.08 was applied.

**Figure 4:**
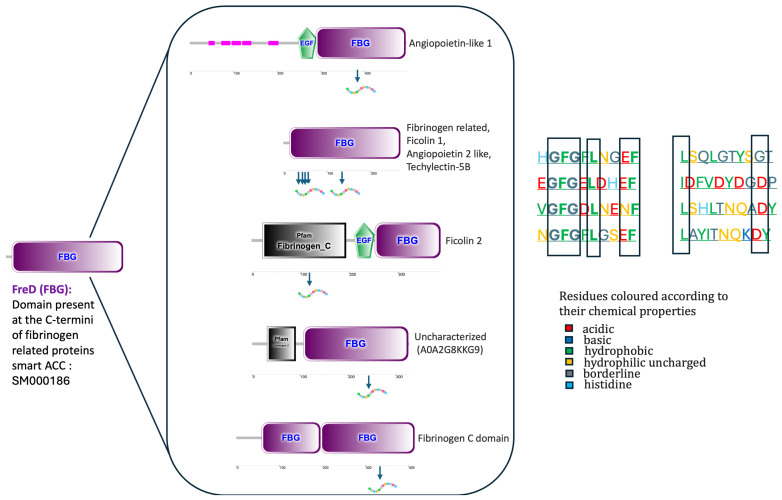
SMART analysis showing FReDs of proteins from which peptides are produced. Peptide sites are shown by arrows. The left part of the figure shows the amino acid composition of protein residues according to their chemical properties.

We also analyzed the positions of the top-ranked peptides on the corresponding proteins they originate from, aiming to determine whether they fall within conserved protein domains, using the SMART web tool. Indeed, most of the peptides are in conserved domains. Results of SMART analysis for all the top 40 peptides are in the Supplementary Materials. SMART analysis showed that eight of the top-ranked peptides fall into FReDs of fibrinogen-related proteins, mainly in the C-terminal portion, confirming the STRING investigation. Analyzing their residue composition, two groups were identified: a first one rich in basic and hydrophobic residues and fewer acidic residues, mainly enriched in the amino acids glycine (G), phenylalanine (F) and glutamic acid (E). A second group was rich in hydrophobic and acidic residues and mainly enriched in the amino acids leucine (L) and aspartic acid (D), as shown in Figure 4.

As previously shown, the peptides that have been investigated exhibit anti-inflammatory and antioxidant features. Both these features have been linked to properties of the amino acid composition, such as the charge, the polarity, and basic or acidic composition.

They can neutralize free radicals and are more potent than their parent proteins, especially in marine peptides. The mechanisms behind their antioxidant effects include hydrogen donation, free radical scavenging, and metal ion chelation. Specific amino acids like leucine (L), phenylalanine (F), aspartic acid (D), alanine (A), and histidine (H) enhance this activity, as shown by [45,46]. Thus, they could act in different ways, such as ROS inactivation, quenching free radicals, chelating prooxidative transition metals, and donating electrons/hydrogen.

Moreover, there is evidence that antioxidant peptides are histidine- and hydrophobic-rich residues. This feature is important because hydrophobic amino acids have higher partition coefficients than hydrophilic ones and can more easily cross through the lipid bilayer’s hydrophobic core, thereby increasing membrane permeability. Peptides having tyrosine residues at both the C- and N-terminal positions could also exert antioxidant effects [47,48]. Some of them also have acidic residues, such as glutamic acid (E) and aspartic acid (D).

With regard to the anti-inflammatory properties of peptides linked to amino acid composition, it is known that positively charged amino acids in peptides might activate chemokine receptors, leading to an anti-inflammatory response. Anti-inflammatory peptides often contain glutamine and arginine and hydrophobic and positively charged amino acids (such as valine, histidine, and lysine). This evidence strengthens the hypothesis of peptides having a potentially relevant role in regulating the response to ROS linked to oxidative stress and the modulation of inflammatory pathways.

Finally, for this cluster of eight peptides, we also performed molecular dynamics analysis of known significant targets with inflammatory and oxidant activity. Molecular dynamics allows us to determine if these potentially relevant peptides linked to fibrinogen-related proteins can interact with relevant protein targets linked to inflammation and oxidative processes. The analysis was performed, respectively, by having as inflammatory targets four of the major pleiotropic proteins involved in pathways of inflammation processes, namely TNF-α, NF-κB, TGF-β, and Keap-1, and iNOS, NOX-2 and myeloperoxidase as protein targets involved in oxidative stress.

**Figure 5:**
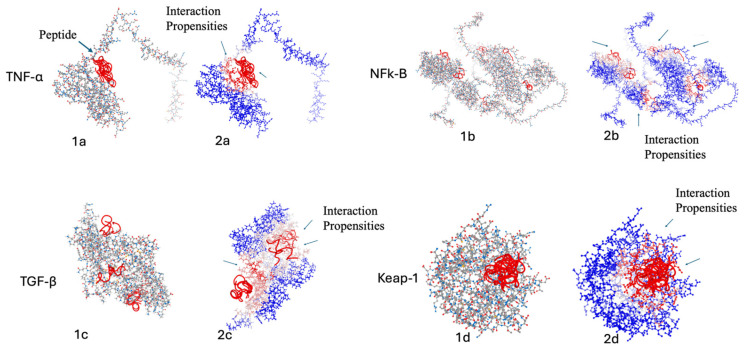

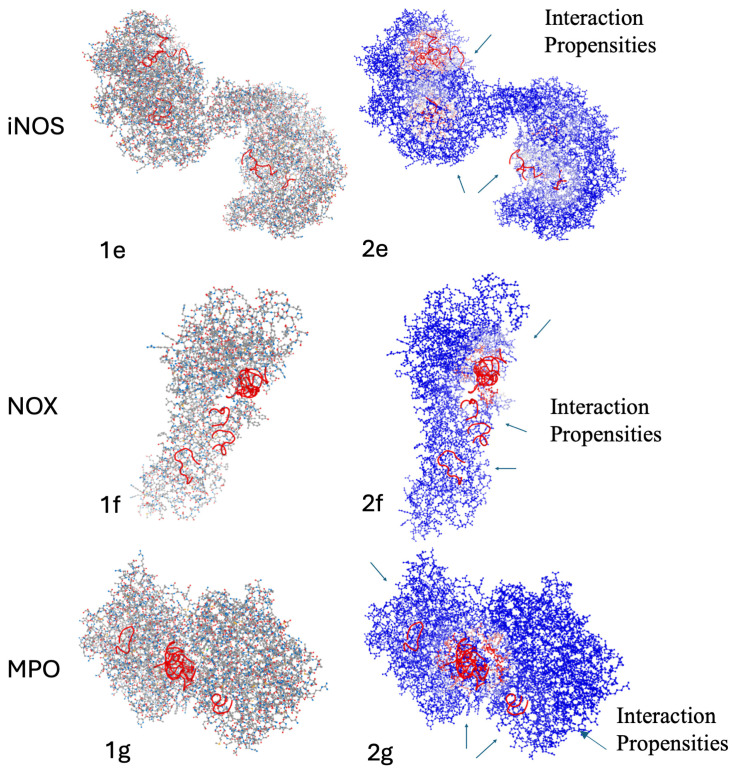
Molecular dynamics of *H. tubulosa* VGFGDLNENF peptide (red colored) and *H. sapiens* (gray/blue colored). (**a**) Interaction analysis with TNF-α protein, (**b**) interaction analysis with NFK-b protein, and (**c**) interaction analysis with TGF-β protein and (**d**) with Keap-1 for anti-inflammatory pathways; (**e**) interaction analysis with iNOS protein, (**f**) interaction analysis with NOX2 protein and Meloperoxidase (**g**) for antioxidant pathways.

**Figure 6:**
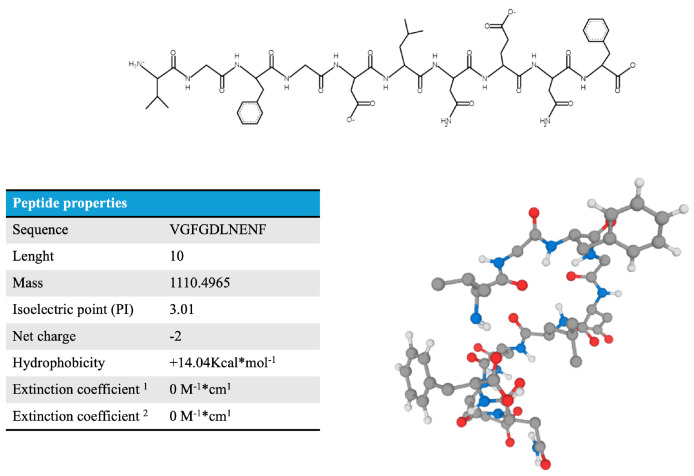
Physicochemical properties and folding structure of *H. tubulosa* VGFGDLNENF. The figure represents the 2D structure of the peptide VGFGDLNENF. The 2D structure was obtained by using PepDraw tool (https://www2.tulane.edu/~biochem/WW/PepDraw/) (accessed on 10 January 2026). Different chemical properties are shown in figure: the sequence of the peptide in amino acid composition; the mass, which is the sum of monoisotopic masses of all amino acid residues in the peptide; the isoelectric point, which is the pH at which the net charge of a peptide is zero. The calculation of the pI assesses the partial charge of the peptide at various pH values starting from 0 and incrementing by 0.01 pH units per step. Then the Net charge is reported, that is, the sum of positively (basic) and negatively (acidic) charged residues at neutral pH. The hydrophobicity here is the free energy associated with transitioning a peptide from an aqueous environment to a hydrophobic environment like octanol. Finally, the molar extinction coefficient is shown. It is a factor used to describe how much light a molecule absorbs. In this case the light is at a wavelength of 280 nm. The most strongly absorbing side chains at this wavelength are tryptophan, tyrosine, and cysteine when it forms disulfide pairs called cystines. The molar extinction is calculated by first counting the number of tryptophans (W), the number of tyrosines (Y), and the number of disulfide bonding pairs (cystines). Then the following formula is used to calculate the first extinction coefficient: W × 5500 + Y × 1490 + cystines × 125. This calculation assumes that all cysteines pair into cystines. The second version assumes that all cysteines are reduced and there are no cystines, thus it is calculated as W × 5500 + Y × 1490.

As an example of peptide–target molecular dynamics, the first-ranked peptide is shown in Figure 5 interacting with different proteins of the *H. sapiens* species, which are key components in the inflammation pathway and oxidative stress. Figure 5 shows the best 10 models of peptide–protein molecular modeling between the VGFGDLNENF peptide identified from the putative ficolin protein in the Holothuria proteome and the TNF-α, NFK-b, TGF-β, iNOS, NOX2 and myeloperoxidase proteins of Homo sapiens, and VGFGDLNENF peptide properties and its folding structure are shown in Figure 6.

Part 1 and part 2 of the figure represent, respectively, (1) the best 10 predicted interaction sites between the peptide and the target protein, and (2) the same peptide–target interaction evidencing the protein interaction propensities. Balls and Stick representation shows the target protein, and the red-colored cartoons show the different peptides interacting with the target protein located in different interaction sites. Analysis was performed through the PEP-SiteFinder tool 1.0.

## 3. Discussion

In the last decade, growing attention has been oriented toward natural sources for the discovery of compounds with antioxidant and anti-inflammatory features, particularly those extracted from marine organisms [20,21,22]. Among these bioactive molecules, peptides have gained significant interest due to their diverse biological functions and therapeutic potential. Marine-derived peptides have been recognized as key modulators of both oxidative stress and inflammatory processes through a variety of complex and interconnected mechanisms.

These peptides can influence inflammation by reducing the production of pro-inflammatory mediators. In addition to directly scavenging free radicals, certain peptides are capable of binding metal ions, thereby limiting ROS generation and enhancing endogenous antioxidant defense systems [49]. Furthermore, they can interfere with major signaling pathways involved in the inflammation process, such as nuclear factor-kappa B (NF-κB), tumor necrosis factor-α (TNF-α), and the Keap1/Nrf2 regulatory axis.

To explore these properties, we implemented a bioinformatics-driven strategy supported by artificial intelligence (AI)-based tools to identify candidate peptides with potential antioxidant and anti-inflammatory activities. These peptides were derived from proteins extracted from both cellular and humoral fractions of the coelomic fluid of *H. tubulosa*, previously characterized through mass spectrometry analysis. The integration of omics data with computational approaches enables a substantial reduction in experimental costs and time, while accelerating candidate selection and prioritization.

From an initial dataset of 174 annotated proteins identified through proteomic analysis, a systematic in silico workflow was applied to generate peptide sequences. Tryptic digestion was simulated to produce peptide fragments, followed by the generation of overlapping sequences using a sliding window approach of 10 amino acids. This process resulted in a large pool of candidate peptides, which were subsequently filtered based on multiple physicochemical and functional criteria to refine the selection.

The screening pipeline included the evaluation of key properties such as predicted bioactivity, intestinal stability, toxicity, and cell membrane permeability. These parameters are essential to identify peptides that are not only biologically active but also stable under physiological conditions, non-toxic, and capable of efficiently crossing cellular membranes. Following this selection process, the antioxidant and anti-inflammatory potential of the shortlisted peptides was further assessed.

The simultaneous evaluation of these two biological activities is particularly relevant, as oxidative stress and inflammation are closely interconnected. Excessive ROS production can trigger inflammatory pathways, while inflammation itself can further enhance oxidative stress, creating a self-perpetuating cycle. Therefore, targeting both processes concurrently represents a promising strategy for therapeutic intervention in chronic diseases. Ultimately, the workflow led to the identification of the top 40 peptide candidates.

Functional annotation of the source proteins revealed that many peptides originated from extracellular matrix-associated proteins, including fibrinogen, filamin-B, and calmodulin. Additional peptides were derived from proteins involved in signaling pathways, such as Ras-related proteins, as well as proteins associated with metabolism (e.g., transferrin) and immune responses (e.g., ficolin-2), as supported by STRING analysis.

Among the identified peptides, particular attention was given to those derived from thymosin-β, which is known to reduce the production of superoxide and nitric oxide radicals in oxidative stress conditions [50]. This protein also contributes to the attenuation of inflammatory responses by inhibiting NF-κB signaling and modulating the expression of different cytokines, stimulating the inflammation process, such as TNF-α and IL-1β. Additionally, thymosin-β has been linked to anti-apoptotic effects through the regulation of ROS levels and the modulation of key apoptotic markers, including the Bax/Bcl-2 ratio and caspase-3 activity [51].

Melanotransferrin (MTf) was another protein of interest due to its involvement in iron metabolism and redox regulation. Its role in ferroptosis, a form of regulated cell death driven by oxidative imbalance, has been increasingly recognized, particularly in cancer biology [52]. Similarly, filamin-B, known for its role in cytoskeletal organization, has also been implicated in maintaining redox homeostasis through interactions with signaling molecules such as Rac1, a component of the NADPH oxidase complex [53]. These observations are in accord with previous findings indicating that ROS can act both as regulators and effectors of small GTPase signaling pathways, including those involving Ras proteins [54].

Moreover, ROS have been shown to participate in angiopoietin-mediated signaling processes, influencing endothelial cell migration and angiogenesis through NADPH oxidase-dependent mechanisms [55]. Angiopoietins also play protective roles by reducing oxidative stress and limiting inflammation, for instance by downregulating ROS-induced expression of adhesion molecules such as VCAM [56,57]. Calmodulin (CaM) represents another key component in redox signaling, forming a bidirectional regulatory network with ROS that influences calcium-dependent signaling pathways [58].

To further characterize the identified peptides, their positions within the parent proteins were analyzed using the SMART tool to determine their localization within conserved domains. The results indicated that a significant proportion of the top-ranked peptides were located within conserved regions, particularly within fibrinogen-related domains (FReD), predominantly in the C-terminal region. These domains are widely distributed among fibrinogen-like proteins, which are known to be susceptible to oxidative modifications. Fibrinogen plays a critical role in coagulation, inflammation, tissue repair, and cellular interactions, all of which can be influenced by oxidative stress. Previous studies have suggested that its amino acid composition, particularly the presence of methionine residues, may contribute to ROS scavenging activity [59].

Based on these observations, we further investigated whether the amino acid composition of peptides derived from FReD domains could be associated with their predicted biological activities. Two main groups of peptides were identified: one enriched in basic and hydrophobic residues, and another characterized by higher proportions of hydrophobic and acidic amino acids. Specific residues such as glycine, phenylalanine, glutamic acid, leucine, and aspartic acid were particularly abundant. It has been reported that aromatic and acidic amino acids can act as electron or proton donors, thereby neutralizing free radicals [60]. In addition, sulfur-containing amino acids, including cysteine and methionine, are known for their radical-scavenging properties and their role in modulating inflammatory responses [61,62].

The presence of hydrophobic and histidine-rich sequences has also been associated with enhanced antioxidant activity, while tyrosine residues located at terminal positions may further contribute to radical scavenging [47,48]. These characteristics are consistent with previous findings indicating that marine-derived peptides often exhibit stronger antioxidant activity compared to those from other sources [63,64].

Metal chelation represents another important mechanism underlying antioxidant activity. Amino acids such as histidine, lysine, arginine, glutamate, and aspartate are known to facilitate the binding of metal ions, thereby preventing metal-catalyzed oxidative reactions [65,66,67]. Additionally, positively charged residues may contribute to anti-inflammatory effects by interacting with chemokine receptors and modulating immune responses [68,69,70]. At the same time, negatively charged residues and specific amino acids such as glutamine and arginine have also been associated with anti-inflammatory properties [71].

Most bioactive peptides described to date are relatively small, typically consisting of 2–40 amino acids and having molecular weights below 3 kDa [72]. Another critical factor influencing peptide efficacy is membrane permeability, which is largely determined by hydrophobicity. Peptides containing hydrophobic residues are more likely to penetrate lipid bilayers, thereby enhancing their biological activity [73].

To complement these analyses, molecular modeling studies were conducted to investigate the interaction between selected peptides and key protein targets involved in inflammation and oxidative stress. These included TNF-α, NF-κB, TGF-β, and Keap1 for inflammatory processes, as well as inducible nitric oxide synthase (iNOS), NOX-2, and myeloperoxidase for oxidative stress.

TNF-α and NF-κB are central mediators of inflammation, with TNF-α acting as a pro-inflammatory cytokine and NF-κB functioning as a transcription factor regulating the expression of inflammatory genes [74,75,76]. TGF-β exhibits a dual role, depending on the cellular context. Keap1, on the other hand, regulates the Nrf2 pathway by promoting Nrf2 degradation under basal conditions, thereby controlling antioxidant responses [77]. Disruption of the Keap1–Nrf2 interaction enables Nrf2 activation, leading to the expression of cytoprotective genes such as HO-1 and NQO1, which contribute to the reduction in oxidative stress and inflammation [78].

Among oxidative stress-related targets, myeloperoxidase represents a major enzymatic source of ROS in immune cells [79], while iNOS and NOX-derived ROS are key contributors to oxidative damage in various pathological conditions [80,81]. NOX-2 plays a critical role in ROS production during the respiratory burst in phagocytic cells and is closely associated with inflammatory responses [82].

In summary, the regulation of inflammation and oxidative stress is essential for maintaining cellular homeostasis. While moderate levels of ROS are involved in signaling processes, excessive accumulation leads to cellular damage and disease progression. Therefore, the development of bioactive peptides capable of modulating ROS levels represents a promising therapeutic strategy. The combination of marine-derived peptide discovery with AI-based computational approaches provides an efficient platform for identifying novel candidates with potential applications in the treatment of inflammation- and oxidation-related diseases.

## 4. Materials and Methods

### 4.1. Animals

Sixty healthy adult *H. tubulosa* specimens (length 11 ± 0.98 cm and body weight 46 ± 7.5 g) were collected in the Gulf of Palermo (Sicily, Italy). They were acclimatized for 1 week in the laboratory by using a constantly aerated aquarium (temperature of 15 ± 2 °C) and were fed a commercial invertebrate feed until 24 h before sampling (Algamac 3000, Aquafauna BioMarine Inc., Hawthorne, CA, USA).

### 4.2. Sample Preparation

Coelomic fluid (CF) was collected by making a 3–5 cm incision on the antero-dorsal side of each specimen using a scalpel. In order to prevent clotting and maintain cellular integrity, the fluid, which contained both humoral and cellular components, was allowed to percolate into 50 mL Falcon tubes that had been filled previously with Ethylenediamine tetra-acetic acid (ISO-EDTA) anticoagulant (20 mM Tris, 0.5 mM NaCl, 70 mM EDTA; pH 7.5). To separate the cell pellet from the humoral component (cell-free coelomic fluid), CF was centrifuged at 1000× *g* for 10 min at 4 °C after being stored on ice. In a 1X RIPA buffer with 1:200 antiprotease added, the cell pellet was sonicated. The cell-free coelomic fluid and the resultant supernatant (cell pellet lysate) were frozen at −80 °C and lyophilized following centrifugation at 20,000× *g* for 10 min at 4 °C. After that, all the cell lysates were combined to create a single sample that had enough protein for analysis. The cell-free coelomic fluids underwent the same process. While internal proteins extracted from coelomocytes are found in the cellular component, extracellular proteins secreted by coelomocytes are represented by proteins in the humoral fraction.

### 4.3. Proteome

For mass spectrometry analysis, see [83].

### 4.4. Peptide Bioinformatics Analysis Workflow

Mass spectrometry analysis of *H. tubulosa* specimens identified 174 unique annotated proteins from the humoral component (cell-free coelomic fluid) and 349 unique peptides from the cellular component (cell pellet) of the coelomic fluid. A bioinformatic approach was subsequently applied to select distinct peptides derived from the protein data using tryptic peptides. This process enabled the generation of computational peptides with a 10-amino-acid window from the identified protein sequences. These computationally derived peptides were filtered through in silico analyses based on various peptide features to rationalize candidate selection from the extensive proteomics dataset. The proposed bioinformatic workflow included several steps: evaluation of peptide bioactivity, assessment of toxicity, analysis of peptide half-life in an intestinal environment, and a permeability test. Finally, antioxidative and anti-inflammatory properties were evaluated (Figure 1).

### 4.5. Bioinformatics Analysis

#### 4.5.1. Peptides Bioactivity Evaluation

PeptideRanker [84] uses a unique N-to-1 neural network to predict bioactive peptides. Users can provide a list of peptides to the web tool, and the peptides are evaluated based on the probability that they are bioactive. It is important to note that this is not an estimate of the bioactivity level. If a peptide’s predicted value is greater than 0.5, it is deemed bioactive. The user may decide to change the threshold using a higher score in order to reduce the number of false positives. We anticipate that selecting a threshold of 0.8 will lower the false positive rate from 11% and 16% at a 0.5 threshold to 2% and 6% at a 0.8 threshold for long and short peptides, respectively, based on our tests (see article). However, increasing the threshold to 0.8 from 0.5 also reduces the true positive rate. The user needs to choose a threshold carefully based on their needs (http://distilldeep.ucd.ie/PeptideRanker/) (accessed on 10 January 2026).

#### 4.5.2. Prediction of Half-Life of Peptides

The half-life of peptides (HLP) tool [85] (https://webs.iiitd.edu.in/raghava/hlp/) (accessed on 10 January 2026) allows the prediction of the half-life of peptides in an intestine-like environment. For every peptide, all possible mutants are generated (single mutation at each position per cycle), and a prediction of half-life and physicochemical properties (e.g., charge, polarity, hydrophobicity, volume, pK) of mutant peptides is calculated. Models used in this server were trained on peptides whose half-life is experimentally determined in crude intestinal protease preparations. Different possibilities of analysis for each peptide are returned, such as the design of stable peptides, the scanning of peptides in a known protein, and the analysis of multiple peptides to predict/compute half-life and physicochemical properties. The minimum length for a peptide to be analyzed is 5 amino acids. It is important to note that one of the major problems in designing peptide-based drugs is their short half-life or stability in intestinal proteases. Peptides are highly sensitive to degradation by the action of proteolytic enzymes and rapidly cleared from the body before performing/showing the desired effect [85].

#### 4.5.3. Peptides Toxicity Prediction

ToxinPred3.0 is an improved version of ToxinPred for peptide toxicity prediction [86]. It uses a sizable dataset of experimentally verified toxic and non-toxic peptides (5518 toxic and 5518 non-toxic peptides) and is based on machine learning and deep learning techniques. It uses a variety of data and prediction methods, such as MERCI, deep learning, and machine learning [87]. (https://webs.iiitd.edu.in/raghava/toxinpred3/) (accessed on 10 January 2026). After receiving fasta peptide sequences, the prediction module generates a toxicity prediction. Several machine learning models are employed, including extra tree (ET), ANN with LSTM in the deep learning neural network model, and their hybrid techniques (ET + MERCI and DL + MERCI). One or more models can be used in combination to make the forecast. The ML + MERCI hybrid or ensemble strategy, which is the server set as the default method, appears to be the most accurate in our assessments. This final ML model was used for toxicity prediction analysis. The toxicity value of peptides is predicted using a variety of metrics, including ML score, MERCI score, hybrid score, and PPV. The MERCI motif-based method makes it possible to identify patterns that are unique to both toxic and non-toxic peptides. Every forecast has a weight of ‘+0.5’ for potentially toxic peptides (positive predictions), ‘−0.5’ for non-toxic peptides (negative predictions), and ‘0’ for cases in which no matches are identified. This weight assignment provides a quantitative measure of confidence in each prediction. In the hybrid approach, the scores obtained from both the MERCI method and the machine learning or deep learning scores are combined to calculate an overall score. The ToxinPred tool also uses positive predictive value (PPV) calculation for all the predictions. The positive predictive value represents the probability that a positive prediction (indicating toxicity) accurately indicates that the peptide is truly toxic.

### 4.6. Membrane Permeability Test

The capacity of particular peptides to pass across the cell plasma membrane was tested using the Cell-Penetrating Peptides service [88] (https://biomembhub.org/cellpm/) (accessed on 10 January 2026). PerMM—A Web Tool and Database for Analysis of Passive Membrane Permeability—includes it. In consideration of the cell’s physiological state, the following environmental parameters were established: pH of 7.4 and temperature of 310 K. Fasta format was used to enter the input files. The energy transfer values associated with the chemicals’ capacity to cross the cell membrane were displayed in the analysis’s result, which was also displayed in three dimensions.

### 4.7. Prediction of Anti-Inflammatory Properties of Peptides

AIPpred [89] is a web-based prediction server for anti-inflammatory peptides (http://www.thegleelab.org/AIPpred/) (accessed on 10 January 2026). An optimal DPC was used as an input feature for the development of the Random Forest (RF)-based prediction model. AIPpred predicts for each peptide its class and probability values. Its methodology is based on a four-step workflow. First, the dataset is built by using the construction of the benchmarking and independent datasets; then, there is a feature extraction from the primary sequences, including amino acid composition, atomic composition, chain-transition-composition, dipeptide composition, and physicochemical properties; third, a RF algorithm is used to compute a feature importance score (FIS); and finally, a model with the best performances and AUC is considered as the final model, and the corresponding features constitute the optimal feature set. Due to the imbalanced dataset used to train the model, the optimal probability cut-off value of 0.36 was chosen via grid search for AIPpred to define the class. This implies that a peptide will be classified as anti-inflammatory if the probability value is greater than 0.36.

### 4.8. Prediction of Antioxidative Properties of Peptides

AnOxPePred [90] predicts the antioxidative (free radical scavenging (FRS) and ion chelating (Chel)) properties of peptides through the use of a convolutional neural network (https://services.healthtech.dtu.dk/service.php?AnOxPePred-1.0) (accessed on 10 January 2026). Both scavenger and chelator activities are predicted. The input of the prediction tool is a fasta sequence of protein or peptide. The “peptide mode” of analysis is selected. A standard limit of 2–30 residues is used for every peptide prediction. A curated dataset consisting of experimentally tested antioxidant and non-antioxidant peptides was used to train the model. For a variety of metrics, the AnOxPePred method displays a prediction performance better than a k-NN sequence identity-based approach [90]. In the benchmark dataset used to train the algorithm, each peptide was binary labeled for the two classes, free radical scavenger (FRS) and chelator. The classes were labeled 1 (positive) if their source had measured/indicated an activity and 0 (negative) otherwise. So, the FSR and Chel scores range between 0.9 and 0.1 ratios.

After this first step of the bioinformatics workflow, the top 40 peptides were all identified as having high scores for all the features analyzed in the analysis workflow. For this list of top-ranked peptides, the following in silico analyses were then performed.

### 4.9. Protein Annotation

Peptide protein annotation was performed by using the Uniprot web tool [91] (https://www.uniprot.org/) (RRID:SCR_002380) (accessed on 10 January 2026). This web tool allows ID mapping (https://www.uniprot.org/id-mapping) (accessed on 10 January 2026) for protein sequence and functional information.

### 4.10. Protein–Protein Network Analysis

STRING [92] is a database of known and predicted protein–protein interactions. The interactions are both direct (physical) and indirect (functional); they come from different sources, such as computational prediction, knowledge transfer between organisms, and interactions aggregated from other (primary) databases. It has different additional functionalities, such as clustering analysis, which is performed through the K-mer algorithm, and functional analysis.

### 4.11. Protein Domains Analysis

Protein domains can be identified and annotated using SMART v10 [93], a Simple Modular Architecture Research Tool. Phyletic distributions, functional class, tertiary structures, and functionally significant residues are used to identify several domain families that are present in signaling, extracellular, and chromatin-associated proteins. Within designated taxa, proteins with particular domain combinations can be found.

### 4.12. Peptide–Protein Interaction Prediction Through Dynamic Modeling

PEP-SiteFinder [94] is a tool that allows the identification of protein residues predicted to be at the peptide–protein interface, given the structure of a protein and the sequence of a peptide. It is based on the 3D de novo generation of peptide conformations given its sequence. It also returns a propensity index, which is indicative of the confidence of the prediction. (http://bioserv.rpbs.univ-paris-diderot.fr/PEP-SiteFinder; https://doi.org/10.1093/nar/gku404) (accessed on 10 January 2026). There are different inputs requested by the web tool, such as the PDB structure of the protein target and the sequence of the peptide under investigation. There are no limits on the protein size, but the peptide sequence must be less than 36 amino acids. The PEP-SiteFinder flowchart consists of different steps. First, the PEP-FOLD tool [95] is used to predict an ensemble of conformations from the peptide sequence, independently of the protein. Then, for each peptide structure that is produced by the tool, systematic rigid docking is performed using the ATTRACT docking protocol [96]. Finally, the propensities of protein residues are assessed to interact with the peptide. To perform dynamic modeling, the peptide sequence and the PDB file of the target proteins were downloaded into the web tool. The target proteins used were the inducible nitric oxide synthase (iNOS), NADPH Oxidase 2 (NOX2) and myeloperoxidase molecules for the antioxidant analysis, and the TNF-α, TGF- ß, NFk-B and Keap-1 for the anti-inflammatory analysis. PDB files were downloaded from the UniProt database (https://www.uniprot.org/) (accessed on 10 January 2026).

## 5. Conclusions and Further Directions

Over the past decade, research on marine organisms has identified numerous antioxidants and anti-inflammatory compounds, particularly peptides, which are pivotal in mitigating inflammation and oxidative stress. These peptides can regulate inflammatory responses by suppressing pro-inflammatory cytokines and reactive oxygen species (ROS), utilizing mechanisms such as scavenging activity and ion chelation to enhance the body’s antioxidant defenses. A bioinformatics approach combined with mass spectrometry yielded the 40 top peptides, including peptides that fall into FreD domains, which are related to their protective functions against oxidative damage. The identified peptides specifically bind key target proteins involved in inflammation and oxidative processes, inhibiting them and leading to the inhibition of signaling cascades linked to these pathways. Understanding the interplay between oxidative stress and inflammation can lead to more effective therapeutic strategies for chronic diseases by targeting both processes concurrently.

Of course, this is just a preliminary study for the identification of the antioxidant and anti-inflammatory activity of peptides extracted from marine organisms. We will further address our study on the experimental validation of the identified peptides to confirm their predicted antioxidant and anti-inflammatory activities, also with cell-based models by measuring key mediators such as cytokines, nitric oxide, and inflammatory enzymes. Further investigations should explore the underlying mechanisms of action, particularly their effects on major signaling pathways such as NF-κB and Nrf2/Keap1 using molecular and biochemical techniques.

## Figures and Tables

**Table 1:** Top 40 anti-inflammatory and antioxidant peptides derived from bioinformatic analysis: cell pellet/cell-free indicate the source of *H. tubulosa* coelomic fluid (humoral component (cell-free coelomic fluid) and the cellular component (cell pellet)); then, the Uniprot ID indicates the Uniprot accession number identifier. The bioactivity score indicates the probability of a peptide being bioactive based on a neural network algorithm. The higher the score, the higher the probability of a peptide being bioactive. Toxicity prediction was used to determine whether a peptide was toxic or non-toxic. For the anti-inflammatory probability, the optimal probability cut-off value of 0.36 was chosen via a grid search for AIPpred to define the class of anti-inflammatory peptide (AIP) or non-AIP (non-AIP). This implies that a peptide will be classified as anti-inflammatory if the probability value is greater than 0.36. FSR AnOx and Chel AnOx refer to the free radical scavenging (FSR) and ion chelating (Chel) properties of peptides, and their scores range between 0.9 and 0.1. If the half-life (s) parameter is >0.1, the stability is low; if the half-life (s) parameter is between 0.1 and 1.0 the stability is normal; and if the half-life (s) parameter is >1.0 the stability is high.

Sequence	Cell Pellet/Uniprot ID Cell Free		Name	Bioactivity	Toxicity	Anti-Inflammatory		FSR AnOx	Chel AnOx	Half Life	
					Prediction	AIP or Non-AIP	Prob			Half-Life [s]	Stability
VGFGDLNENF	cell free	A0A2G8L651_STIJA	Ficolin-1	0.83	Non-Toxin	AIP	0.43	0.47		6.29	High
NGFGFLNTEF	cell free	A0A2G8K011_STIJA	Fibrinogen-like	0.73	Non-Toxin	AIP	0.41	0.46		5.58	High
HGFGFLNGEF	cell free	A0A2G8K2I6_STIJA	Fibrinogen C-terminal domain-containing protein	0.73	Non-Toxin	AIP	0.39	0.45		5.22	High
NGFGFLGSEF	cell free	A0A2G8JM67_STIJA	Putative ficolin-2-like	0.69	Non-Toxin	AIP	0.50	0.45		4.72	High
FGTIGFDDGI	cell pellet	A0A2G8LDJ7_STIJA	Alkaline phosphatase	0.56	Non-Toxin	AIP	0.42	0.40		3.92	High
DSYVGDEAQS	cell free	Q26378_TRIGR	CyI actin	0.56	Non-Toxin	AIP	0.38	0.33	0.23	3.90	High
LFDSAGYTDS	cell free	A0A0K1Z5K0_HOLGL	Melanotransferrin 2	0.53	Non-Toxin	AIP	0.39	0.40		3.69	High
DSNFGINDLE	cell pellet	A0A0K1Z5K0_HOLGL	Melanotransferrin 2	0.41	Non-Toxin	AIP	0.38		0.24	3.37	High
ESINVEQAFQ	cell pellet	A0A2G8LEX4_STIJA	Putative ras-related protein	0.37	Non-Toxin	AIP	0.52	0.32	0.25	3.34	High
EGFGELDHEF	cell free	A0A2G8KAK2_STIJA	Angiopoietin-like 1	0.37	Non-Toxin	AIP	0.42	0.52		3.33	High
NGFGELDHEF	cell free	A0A2G8L6S5_STIJA	Putative ficolin-2-like	0.34	Non-Toxin	AIP	0.38	0.49		3.24	High
NTLPTKDTID	cell free	A7LGB0_HOLGL	Thymosin beta	0.31	Non-Toxin	AIP	0.41		0.22	2.79	High
ADLITLDGGD	cell free	A0A0K1Z5K0_HOLGL	Melanotransferrin 2	0.31	Non-Toxin	AIP	0.45	0.41	0.23	2.76	High
LFDSEDYEGS	cell free	A0A0K1Z4Z0_HOLGL	Melanotransferrin 4	0.30	Non-Toxin	AIP	0.40	0.46		2.75	High
IDFVDYDGDP	cell free	A0A2G8LI29_STIJA	Fibrinogen C-terminal domain-containing protein	0.29	Non-Toxin	AIP	0.38	0.57		2.71	High
YPIEHGIITN	cell free	W4ZD41_STRPU	Uncharacterized protein	0.23	Non-Toxin	AIP	0.45		0.24	2.58	High
DGNGFISAAE	cell free	A0A2G8LBK1_STIJA	Putative calmodulin-like	0.23	Non-Toxin	AIP	0.47		0.250	2.57	High
ETDSEEEIRE	cell pellet	A0A2G8LBK1_STIJA	Putative calmodulin-like	0.21	Non-Toxin	AIP	0.43		0.23	2.55	High
ASLPVEFTVD	cell pellet	A0A2G8KHF6_STIJA	Putative filamin-B	0.18	Non-Toxin	AIP	0.39	0.37	0.20	2.59	High
KEEIEGELSN	cell pellet	A0A2G8KVL2_STIJA	Uncharacterized protein	0.18	Non-Toxin	AIP	0.44	0.34	0.22	2.43	High
AMSIMNSFVN	cell free	H2B_ASTRU	Histone H2B, gonadal	0.17	Non-Toxin	AIP	0.37	0.35	0.20	2.43	High
LAYITNQKDY	cell pellet	A0A2G8LHI5_STIJA	Techylectin-5B	0.16	Non-Toxin	AIP	0.46	0.39		2.42	High
PSSESSIQDD	cell free	A0A2G8KCF3_STIJA	Putative solute carrier family 15member 4-like	0.16	Non-Toxin	AIP	0.41	0.27	0.26	2.21	High
YPIEHGIVTN	cell free	Q26378_TRIGR	CyI actin	0.15	Non-Toxin	AIP	0.38	0.46	0.22	2.17	High
TSGWIVPIGL	cell free	A0A0K1Z5K0_HOLGL	Melanotransferrin 2	0.15	Non-Toxin	AIP	0.48	0.44	0.22	2.07	High
LSISTLEDTI	cell free	W4XW27_STRPU	Uncharacterized protein	0.13	Non-Toxin	AIP	0.47	0.28	0.23	2.07	High
VFDKDGNGFI	cell free	A0A2G8LBK1_STIJA	Putative calmodulin-like	0.13	Non-Toxin	AIP	0.40	0.38	0.21	2.07	High
GIHVPGSPFQ	cell pellet	A0A2G8JE62_STIJA	Putative filamin-B	0.12	Non-Toxin	AIP	0.38	0.40		2.06	High
ATAEEGAEFL	cell free	A0A2G8KN50_STIJA	Putative nucleolin	0.11	Non-Toxin	AIP	0.43	0.42		2.04	High
SLGQNPTEAE	cell pellet	A0A2G8LBK1_STIJA	Putative calmodulin-like	0.11	Non-Toxin	AIP	0.47	0.44	0.27	2.00	High
FEVDVQSDGR	cell pellet	A0A2G8KRL6_STIJA	Protein singed	0.11	Non-Toxin	AIP	0.40	0.31	0.20	1.99	High
VISVGTTENK	cell free	C4TQH8_STIJA	Major yolk protein 2	0.11	Non-Toxin	AIP	0.43	0.35	0.21	1.96	High
EAADVIAESP	cell pellet	A0A2D3HXS4_STIJA	Stomatin	0.10	Non-Toxin	AIP	0.44		0.23	1.92	High
LSHLTNQADY	cell free	A0A2G8KZU0_STIJA	Putative angiopoietin-2-like	0.10	Non-Toxin	AIP	0.40		0.23	1.89	High
EAFTDADNFG	cell pellet	W4XKI6_STRPU	Phospholipid scramblase	0.08	Non-Toxin	AIP	0.42	0.46		1.85	High
EGGDEAIIAQ	cell pellet	W4ZL48_STRPU	Helicase C-terminal domain-containing protein	0.08	Non-Toxin	AIP	0.47		0.27	1.79	High
TAGWNIPVGY	cell free	A0A0K1Z5K0_HOLGL	Melanotransferrin	0.06	Non-Toxin	AIP	0.47	0.54		1.77	High
LSQLGTYSGT	cell free	A0A2G8KKG9_STIJA	Uncharacterized protein	0.05	Non-Toxin	AIP	0.37	0.44		1.7	High
EGQADLITLD	cell free	C6KJ79_HOLGL	Melanotransferrin 1	0.05	Non-Toxin	AIP	0.46	0.28	0.21	1.707	High
IGPQHSPNNP	cell pellet	A0A2G8K9B6_STIJA	Putative ras-related protein	0.05	Non-Toxin	AIP	0.44	0.39	0.24	1.696	High

**Table 2:** Peptides annotation. CF and CP indicate, respectively, the source of *H. tubulosa* coelomic fluid (humoral component (cell-free coelomic fluid) and the cellular component (cell pellet)).

Peptide Seq.	CF or CP	Uniprot ID	Protein Name	Species
VGFGDLNENF	cell free	A0A2G8L651	Ficolin-1	*Stichopus japonicus* (*S. japonicus*)
NGFGFLNTEF	cell free	A0A2G8K011	Fibrinogen-like	*S. japonicus*
HGFGFLNGEF	cell free	A0A2G8K2I6	Fibrinogen C-terminal domain-containing protein	*S. japonicus*
NGFGFLGSEF	cell free	A0A2G8JM67	Putative ficolin-2-like	*S. japonicus*
FGTIGFDDGI	cell pellet	A0A2G8LDJ7	Alkaline phosphatase	*S. japonicus*
DSYVGDEAQS	cell free	Q26378	CyI actin	*Tripneustes gratilla* (*T. gratilla*)
LFDSAGYTDS	cell free	A0A0K1Z5K0	Melanotransferrin 2	*Holothuria glaberrima* (*H. glaberrima*)
DSNFGINDLE	cell pellet	A0A0K1Z5K0	Melanotransferrin 2	*Holothuria glaberrima* (*H. glaberrima*)
ESINVEQAFQ	cell pellet	A0A2G8LEX4	Putative ras-related protein	*S. japonicus*
EGFGELDHEF	cell free	A0A2G8KAK2	Angiopoietin-like 1	*S. japonicus*
NGFGELDHEF	cell free	A0A2G8L6S5	Putative ficolin-2-like	*S. japonicus*
NTLPTKDTID	cell free	A7LGB0	Thymosin beta	*Holothuria glaberrima* (*H. glaberrima*)
ADLITLDGGD	cell free	A0A0K1Z5K0	Melanotransferrin 2	*Holothuria glaberrima* (*H. glaberrima*)
LFDSEDYEGS	cell free	A0A0K1Z4Z0	Melanotransferrin 4	*Holothuria glaberrima* (*H. glaberrima*)
IDFVDYDGDP	cell free	A0A2G8LI29	Fibrinogen C-terminal domain-containing protein	*S. japonicus*
YPIEHGIITN	cell free	W4ZD41	Uncharacterized protein	*Strongylocentrotus purpuratus* (*S. purpuratus*)
DGNGFISAAE	cell free	A0A2G8LBK1	Putative calmodulin-like	*S. japonicus*
ETDSEEEIRE	cell pellet	A0A2G8LBK1	Putative calmodulin-like	*S. japonicus*
ASLPVEFTVD	cell pellet	A0A2G8KHF6	Putative filamin-B	*S. japonicus*
KEEIEGELSN	cell pellet	A0A2G8KVL2	Uncharacterized protein	*S. japonicus*
AMSIMNSFVN	cell free	P02286	Histone H2B, gonadal	*Asterias rubens* (*A. rubens*)
LAYITNQKDY	cell pellet	A0A2G8LHI5	Techylectin-5B	*S. japonicus*
PSSESSIQDD	cell free	A0A2G8KCF3	Putative solute carrier family 15member 4-like	*S. japonicus*
YPIEHGIVTN	cell free	Q26378	CyI actin	*Tripneustes gratilla* (*T. gratilla*)
TSGWIVPIGL	cell free	A0A0K1Z5K0	Melanotransferrin 2	*Holothuria glaberrima* (*H. glaberrima*)
LSISTLEDTI	cell free	W4XW27	Uncharacterized protein	*Strongylocentrotus purpuratus* (*S. purpuratus*)
VFDKDGNGFI	cell free	A0A2G8LBK1	Putative calmodulin-like	*S. japonicus*
GIHVPGSPFQ	cell pellet	A0A2G8JE62	Putative filamin-B	*S. japonicus*
ATAEEGAEFL	cell free	A0A2G8KN50	Putative nucleolin	*S. japonicus*
SLGQNPTEAE	cell pellet	A0A2G8LBK1	Putative calmodulin-like	*S. japonicus*
FEVDVQSDGR	cell pellet	A0A2G8KRL6	Protein singed	*S. japonicus*
VISVGTTENK	cell free	C4TQH8	Major yolk protein 2	*S. japonicus*
EAADVIAESP	cell pellet	A0A2D3HXS4	Stomatin	*S. japonicus*
LSHLTNQADY	cell free	A0A2G8KZU0	Putative angiopoietin-2-like	*S. japonicus*
EAFTDADNFG	cell pellet	W4XKI6	Phospholipid scramblase	*Strongylocentrotus purpuratus* (*S. purpuratus*)
EGGDEAIIAQ	cell pellet	W4ZL48	Helicase C-terminal domain-containing protein	*Strongylocentrotus purpuratus* (*S. purpuratus*)
TAGWNIPVGY	cell free	A0A0K1Z5K0	Melanotransferrin	*S. japonicus*
LSQLGTYSGT	cell free	A0A2G8KKG9	Uncharacterized protein	*Holothuria glaberrima* (*H. glaberrima*)
EGQADLITLD	cell free	C6KJ79	Melanotransferrin 1	*Holothuria glaberrima* (*H. glaberrima*)
IGPQHSPNNP	cell pellet	A0A2G8K9B6	Putative ras-related protein	*S. japonicus*

## Data Availability

The original data presented in the study are included in the article/Supplementary Material; further inquiries can be directed to the corresponding author.

## Supplementary Materials

The following supporting information can be downloaded at: https://www.mdpi.com/article/10.3390/md24050158/s1. Results of SMART analysis for all the top 40 peptides.
